# Nutritional and functional roles of β-mannanase on intestinal health and growth of newly weaned pigs fed two different types of feeds

**DOI:** 10.1093/jas/skae206

**Published:** 2024-07-24

**Authors:** Jonathan T Baker, Zixiao Deng, Adebayo Sokale, Brent Frederick, Sung Woo Kim

**Affiliations:** Department of Animal Science, North Carolina State University, Raleigh, NC 27695, USA; Department of Animal Science, North Carolina State University, Raleigh, NC 27695, USA; BASF Corp, Florham Park, NJ 07932, USA; Cargill Animal Nutrition NA, Minneapolis, MN 55450, USA; Department of Animal Science, North Carolina State University, Raleigh, NC 27695, USA

**Keywords:** growth performance, intestinal health, β-mannanase, nursery pigs

## Abstract

This study aimed to investigate the nutritional and functional roles of β-mannanase on the intestinal health and growth of newly weaned pigs fed a typical or low-cost formulated feeds (LCF). Twenty-four newly weaned pigs at 6.2 kg ± 0.4 body weight (BW) were allotted to three dietary treatments based on a randomized complete block design with sex and initial BW as blocks. Three dietary treatments are as follows: Control, typical nursery feeds including animal protein supplements and enzyme-treated soybean meal; LCF with increased amounts of soybean meal, decreased amounts of animal protein supplements, and no enzyme-treated soybean meal; LCF+, low-cost formulated feed with β-mannanase at 100 g/t, providing 800 thermostable β-mannanase unit (TMU) per kg of feed. Pigs were fed based on a three-phase feeding program for a total of 37 d. On day 37 of feeding, all pigs were euthanized and the gastrointestinal tract was removed for sample collection to analyze intestinal health parameters, mucosa-associated microbiota, and gene expression of tight junction proteins. Pigs fed LCF increased (*P* < 0.05) the relative abundance of Proteobacteria and *Helicobacter* in the jejunal mucosa, tended to decrease (*P* = 0.097; *P* = 0.098) the concentration of malondialdehyde (MDA) and the expression of zona occluden 1 (ZO-1) gene in the jejunum, tended to decrease average daily gain (ADG; *P* = 0.084) and final BW (*P* = 0.090), and decreased (*P* < 0.05) average daily feed intake. Pigs fed LCF + tended to decrease (*P* = 0.088) digesta viscosity, decreased (*P* < 0.05) the relative abundance of *Helicobacter,* and increased (*P* < 0.05) *Lactobacillus* in the jejunal mucosa compared to LCF. Additionally, LCF + tended to increase final BW (*P* = 0.059) and ADG (*P* = 0.054), increased (*P* < 0.05) gain to feed ratio (G:F), and reduced (*P* < 0.05) fecal score compared to LCF. LCF with decreased amounts of animal protein supplements and increased amounts of soybean meal had negative effects on the composition of the mucosa-associated microbiota, intestinal integrity, and growth performance of nursery pigs. Beta-mannanase supplementation to LCF decreased digesta viscosity, increased the relative abundance of potentially health-benefitting microbiota such as *Lactobacillus,* and improved growth and fecal score, thus reflecting its efficacy in low-cost formulated feeds with increased amounts of soybean meal.

## Teaser

Low-cost formulated feeds with high soybean meal fed to nursery pigs increased pathogenic bacteria associated with the jejunal mucosa, impaired intestinal integrity, and decreased growth performance of nursery pigs. Supplementation of β-mannanase decreased digesta viscosity, increased *Lactobacillus* in the jejunal mucosa, reduced diarrhea, and improved growth performance of nursery pigs.

## Introduction

Feed costs constitute between 60% and 70% of the total cost associated with commercial pork production in modern intensive systems and are coupled with increased price volatility of commonly used feedstuffs such as corn and soybean meal (Patience et al., 2015; Langemeier, 2022). Due to the large proportion of capital invested in manufacturing and delivering the proper feeds at the optimal time to the animal, it is important to identify opportunities to reduce feed costs. Nursery feeds typically have the greatest unit cost compared to feeds for pigs in other stages attributed to increased feed complexity with the use of highly digestible feedstuffs, typically of animal origin, to aid the young pigs’ transition from sow milk to solid feeds (Skinner et al., 2014; Jones et al., 2018). Common animal protein sources included in nursery feeds are poultry meal and fish meal, with the latter being highly palatable and possessing a balanced amino acid profile and relatively higher proportions of omega-3 fatty acids (Church and Kellems, 1998; Kim and Easter, 2001). Unfortunately, fish meal production is expected to decline in the future due to constant increases in price and a concomitant reduction in fishery resources (Tacon et al., 2011). Poultry meal is a rendered co-product from poultry harvesting facilities that possesses a crude protein content and amino acid profile similar to fish meal thus welcoming its inclusion in swine feeds (Keegan et al., 2004; Kim et al., 2019a). Unfortunately, the availability of poultry meals for procurement in pig feeds can be limited as the increased vertical integration of the poultry industry has led to poultry meals being commonly included back into the companies’ own poultry feed (Cromwell, 2006).

One of the most commonly used plant protein supplements included in pig feeds is soybean meal (SBM), due to its balanced amino acid profile as well as its ease of procurement. However, the presence of anti-nutritional compounds including allergenic proteins and nonstarch polysaccharides (NSP) limits high inclusion in feeds fed to young animals (Kim et al., 2003; Taliercio et al., 2014). The NSP content of SBM varies from 17% to 27%, and is indigestible for nonruminant animals such as pigs due to a lack of endogenous enzymes for NSP (Choct et al., 2010). Beta-mannan is one such NSP in SBM, with contents ranging from 1.0% to 1.5% in dehulled SBM and 1.1% to 2.1% in nondehulled SBM (Bach Knudsen, 1997; Hsaio et al., 2006). Beta-mannan found in feedstuffs is similar in structure to surface components on the cell wall of certain pathogenic microbes (Kogut, 2022) and identified by a specific mannose receptor, that can stimulate the innate immune system (Peng et al., 1991; Zhang and Tizzard, 1996; Ross et al., 2002), thus eliciting an unwarranted immune response (Arsenault et al., 2017). Intact β-mannan can negatively affect nutrient digestion and increase digesta viscosity, resulting in adverse effects on the immune system and intestinal microbiota of pigs (Kiarie et al., 2013; Jang et al., 2024). The use of β-mannanase in feeds for young pigs could be beneficial by eliminating antinutritional effects of β-mannan especially in LCF with increased amounts of SBM and decreased amounts of animal protein supplements. Serval studies have been recently conducted to investigate the effects of β-mannanase on health and growth of pigs and indicated that β-mannanase could improve growth performance due to the enhance of energy and nutrient utilization (Huntley et al., 2018; Kiarie et al., 2021). In addition, the impact of β-mannanase on immune response of pigs is not consistent, probably because of the variations in feed formulations and sample collections among studies (Huntley et al., 2019; Jang et al., 2024).

Based on previous findings, it is hypothesized that LCF negatively affect mucosal microbiota, intestinal health, and growth performance in nursery pigs. It is also hypothesized that β-mannanase supplementation to LCF mitigates some of the negative effects on mucosal microbiota, intestinal immune responses, and growth performance induced by feeding LCF. Therefore this study aimed to investigate the nutritional and functional roles of β-mannanase on the intestinal health and growth of newly weaned pigs fed a LCF.

## Materials and Methods

The procedure of this study was reviewed and approved by North Carolina State University Animal Care and Use Committee (Raleigh, NC, USA). The experiment was conducted at the North Carolina State University Metabolism Educational Unit (Raleigh, NC, USA).

### Experimental design, animals, and diets

Twenty-four newly weaned pigs (12 barrows and 12 gilts) at 21 d of age with an initial body weight (BW) of 6.2 kg ± 0.4 were individually housed and allotted to three dietary treatments based on a randomized complete block design with sex and initial BW (heavy and light) as blocks. Three dietary treatments consisted of a typical nursery feed including animal protein supplements and enzyme-treated soybean meal (control), a low-cost formulated feed with increased amounts of soybean meal (SBM), decreased amounts of animal protein supplements, and devoid of enzyme-treated soybean meal (LCF), and a low-cost formulated feed with the inclusion of β-mannanase at 100 g/t, providing 800 β-mannanase activity (TMU) per kg of feed (LCF+). Each treatment group had eight replicates with one pig assigned per pen and feeder. Each pen measured 1.50 × 0.74 m, allocating 1.11 m^2^ of pen space per pig. Pigs were fed their respective diets in a four-hole polyethylene nursery feeder with a length of 60 cm, trough depth of 20 cm, and a lip height of 10 cm. All experimental diets met the requirements suggested by NRC (2012) and pigs were fed based on a three-phase feeding program (phase 1: 12 d; phase 2: 9 d; and phase 3: 16 d) for a total of 37 d. The composition of experimental feeds can be seen in Table 1. All experimental diets were in mash and produced at Feed Mill Educational Unit (Raleigh, NC) of North Carolina State University. All experimental diets were sampled and sent to the North Carolina Department of Agriculture and Consumer Services for proximate analysis of nutrient composition (Table 1).

**Table 1.** Composition of phase 1 (5 to 7 kg), phase 2 (7 to 11 kg), and phase 3 (11 to 25 kg) basal diets

	Phase 1 (5 to 7 kg)		Phase 2 (7 to 11 kg)		Phase 3 (11 to 25 kg)	
Item	Control^1^	LCF	Control	LCF	Control	LCF
Feedstuff, %						
Corn, yellow	39.76	35.75	50.06	45.21	67.56	59.97
Soybean meal, 48% crude protein	13.00	28.00	15.00	32.00	20.50	35.00
Whey permeate	23.00	23.00	15.00	15.00	–	–
Hydrolyzed soybean meal^2^	7.00	–	7.00	–	7.00	–
Fish meal	5.00	2.50	4.00	2.00	–	–
Poultry meal	5.00	2.50	5.00	–	–	–
Blood plasma	3.00	3.00	–	–	–	–
Poultry fat	1.30	2.10	1.20	2.50	1.00	1.50
l-Lys HCl	0.43	0.43	0.43	0.43	0.44	0.25
DL-Met	0.20	0.20	0.17	0.18	0.13	0.08
l-Thr	0.15	0.14	0.13	0.13	0.12	0.05
l-Trp	–	–	0.01	–	–	–
l-Val	–	0.03	–	0.03	–	–
Dicalcium phosphate	–	–	0.10	0.07	0.35	0.30
Limestone	0.70	0.90	0.45	1.00	1.15	1.10
Vitamin premix^3^	0.03	0.03	0.03	0.03	0.30	0.30
Trace mineral premix^4^	0.14	0.14	0.14	0.14	–	–
Salt	0.28	0.28	0.28	0.28	0.45	0.45
Supplement^5^	1.00	1.00	1.00	1.00	1.00	1.00
Total	100.00	100.00	100.00	100.00	100.00	100.00
Calculated cost per kg^6^, US dollars	0.92	0.85	0.66	0.61	0.47	0.45
Calculated composition						
DM, %	90.81	90.59	90.34	90.08	89.36	89.36
ME, kcal/kg	3,404	3,402	3,402	3,405	3,361	3,363
Crude protein, %	23.48	23.19	22.07	21.38	19.95	22.06
SID^7^ Lys, %	1.50	1.50	1.35	1.35	1.23	1.23
SID M + C, %	0.83	0.82	0.74	0.74	0.68	0.68
SID Trp %	0.25	0.25	0.22	0.23	0.21	0.24
SID Thr, %	0.89	0.88	0.79	0.79	0.73	0.73
NDF, %	4.68	5.55	5.79	6.74	7.83	8.33
ADF, %	1.84	2.52	2.25	3.01	3.05	3.59
Ca, %	0.86	0.79	0.70	0.70	0.60	0.60
STTD^8^ P, %	0.47	0.39	0.39	0.29	0.22	0.22
Total P, %	0.67	0.59	0.62	0.51	0.45	0.46
Mannan^9^, %	0.28	0.46	0.33	0.53	0.45	0.61
Analyzed composition						
DM, %	89.86	90.14	89.06	88.77	86.99	87.42
Crude protein, %	24.66	24.93	24.58	25.32	21.69	24.46
NDF, %	6.98	7.73	8.01	7.17	9.88	12.69
ADF, %	2.22	3.36	3.49	3.56	4.43	3.90
Ca, %	1.22	1.06	0.97	0.82	0.74	0.74
P, %	0.79	0.66	0.68	0.55	0.50	0.57

^1^Control, control feed.

^2^HP300 (Hamlet Protein Inc., Findlay, OH, USA).

^3^The vitamin premix provided per kilogram of complete diet: 6,614 IU of vitamin A as vitamin A acetate, 992 IU of vitamin D3, 19.8 IU of vitamin E, 2.64 mg of vitamin K as menadione sodium bisulfate, 0.03 mg of vitamin B12, 4.63 mg of riboflavin, 18.52 mg of d-pantothenic acid as calcium panthonate, 24.96 mg of niacin, and 0.07 mg of biotin.

^4^The trace mineral premix provided per kilogram of complete diet: 33 mg of Mn as manganous oxide, 110 mg of Fe as ferrous sulfate, 110 mg of Zn as zinc sulfate, 16.5 mg of Cu as copper sulfate, 0.30 mg of I as ethylenediamine dihydroiodide, and 0.30 mg of Se as sodium selenite.

^5^Supplement includes 0.0 or 0.01% of β-mannanase, supplying 800 TMU/kg; all diets included phytase at 0.01%, supplying 500 phytase unit (FTU)/kg of feed. The remaining amount was added as corn to achieve 1% of the diet. Analyzed value indicates the average phytase activity (FTU/kg) of 589 ± 98 in all feeds and the average β-mannanase activity (TMU/kg) of 592 ± 8 in LCF + feeds; TMU, thermostable mannanase unit; FTU, phytase unit. The analysis of enzyme activity was conducted by BASF Corporation (Florham Park, NJ, USA).

^6^Tentative feed cost is estimated based on the ingredient price at North Carolina State University Feed Education Unit (Raleigh, NC, USA) as of 10/24/22.

^7^SID, standardized ileal digestible.

^8^STTD P, standardized total tract digestible phosphorus.

^9^Mannan content was calculated based on Hsiao et al. (2006).

### Economic analysis

The feed cost and price of feedstuffs were recorded in Raleigh, NC during November 2022. Feed cost per kg of weight gain was calculated as (feed cost/pig)/(weight gain/pig) as previously described by Soleimani et al. (2021).

### Experimental procedures and sample collection

The BW and feed consumption were measured on days 0, 12, 21, and 37 to calculate BW, average daily gain (ADG), average daily feed intake (ADFI), and gain:feed (G:F). Fecal scores were measured every 2 days based on a 1 to 5 scale: 1) very hard and dry feces, 2) firm stool, 3) normal stool, 4) loose stool, and 5) watery stool with no shape following Weaver and Kim (2014).

At the end of day 37 of feeding, all pigs were euthanized by a captive bolt gun followed by exsanguination and removal of the gastrointestinal tract for sample collection. Digesta from the proximal jejunum was collected into Falcon tubes (50 mL), placed on ice, and immediately transferred to the lab to evaluate digesta viscosity. Mid-jejunum segments (3 m after the pyloric duodenal junction) were rinsed with 0.9% saline solution and then fixed in 10% buffered formaldehyde to be used for Ki-67 staining and to evaluate villus height (VH), crypt depth (CD), and villus height to crypt depth ratio (VH:CD). Mucosal samples from the mid-jejunum were scraped by a glass slide and collected in Eppendorf tubes (2 mL), then put it into liquid nitrogen immediately and stored at −80 °C for subsequent immune, oxidative stress, and mucosa-associated microbiota measurements. Mid-jejunal tissue samples were also utilized for measurement of tight junction proteins, claudin-1 (CL-1), occuldins (OC), zona occulden-1 (ZO-1) and mucin (MUC-1 and 2). Each sample was weighed (500 mg), suspended in 1 mL of phosphate-buffered saline (PBS), and homogenized on ice using a tissue homogenizer (Tissuemiser; Thermo Fisher Scientific Inc. Waltham, MA, USA). After homogenization, samples were centrifuged (14,000 × *g* for 3 min) and the supernatant was divided in five aliquots and stored at −80 °C until analyses.

### Digesta viscosity

Following Passos et al. (2015) and Duarte et al. (2019), samples of jejunum digesta from 50 mL tubes were divided into two Falcon tubes (15 mL) and centrifuged at 1,000 × *g* at 4 °C for 10 min to obtain the liquid phase. The liquid phase was then transferred to an Eppendorf tube (2 mL) and centrifuged at 10,000 × *g* at 4 °C for 10 min. The supernatant obtained was transferred to another Eppendorf tube (1.5 mL) for further measurement. Digesta supernatant (0.5 mL) was placed in the viscometer (Brookfield Digital Viscometer, Model DV-II Version 2.0, Brookfield Engineering Laboratories Inc., Stoughton, MA, USA), set at 25 °C. The viscosity measurement was the average between 45.0 s^−1^ and 22.5 s^−1^ shear rates, and the viscosity values were recorded as apparent viscosity in centipoise (cP).

### Relative abundance and diversity of jejunal mucosa-associated microbiota

Mucosal samples from the mid-jejunum were utilized for microbiome sequencing using 16S rRNA gene sequence analysis. Samples were sent to Zymo Research (Irvine, CA, USA) for DNA extraction and microbiome sequencing according to Zymo Research internal protocols. In short, DNA was extracted by Zymo Research using ZymoBIOMICS-96 MagBead DNA Kit (Zymo Research, Irvine, CA, USA). The DNA samples were prepared for targeted sequencing with the Quick-16S Plus NGS Library Prep Kit (Zymo Research, Irvine, CA, USA) and the primer set used was Quick-16S Primer Set V3-V4 (Zymo Research, Irvine, CA, USA). The final PCR products were quantified with qPCR fluorescence readings and pooled together based on equal molarity. The final pooled library was cleaned up with the Select-a-Size DNA Clean & Concentrator (Zymo Research, Irvine, CA, USA), then quantified with TapeStation (Agilent Technologies, Santa Clara, CA, USA) and Qubit (Thermo Fisher Scientific, Waltham, WA, USA). The final library was sequenced on Illumina NextSeq 2000 with a p1 (cat 20075294) reagent kit (600 cycles). The sequencing was performed with 30% PhiX spike-in. Unique amplicon sequences were inferred from raw reads using the Dada2 pipeline (Callahan et al., 2016). Chimeric sequences were also removed with the Dada2 pipeline. Taxonomy assignment was performed using Uclust from Qiime v.1.9.1. Taxonomy was assigned with the Zymo Research Database, a 16S databased that is internally designed and curated, as reference. To initiate the statistical analysis of the microbiota, OTU data were transformed to relative abundance as previously described by Kim et al. (2019b). The OTU with the relative abundance<0.5% within each level were combined as “Other”.

### Intestinal inflammatory status, humoral immune status, and oxidative stress status

Jejunal mucosa samples were weighed (1 g) and suspended in 1 mL of phosphate-buffered saline (PBS) on ice, then homogenized using a tissue homogenizer (Tissuemiser; Thermo Fisher Scientific Inc., Waltham, MA, USA). Following Holanda and Kim (2021), the processed samples were then transferred into a new 2 mL microcentrifuge tube and centrifuged at 14,000 × *g* for 15 min. The supernatant was pipetted into five aliquots and stored at −80 °C. The concentrations of interlukin-6 (IL-6), interlukin-8 (IL-8), tumor necrosis factor alpha (TNF-α), malondialdehyde (MDA), and protein carbonyl were measured by ELISA methods using commercially available ELISA kits according to instructions of the manufacturers. The absorbance was read using an ELISA plate reader (Synergy HT, BioTek Instruments, Winooski, VT, USA) and software (Gen5 Data Analysis Software, BioTek Instruments). The concentration was calculated based on the standard curve created from concentration and absorbance of the respective standard. The concentration of mucosal total protein was determined using the Pierce BCA Protein Assay (23225#, Thermo Fisher Scientific Inc. Rockford, IL, USA) as described by Holanda et al. (2020). The samples were diluted (1:80) in PBS to reach the working range of 20 to 2,000 μg/mL. The absorbance was measured at 562 nm. The concentration of IL-6 in jejunal mucosa was measured by following instructions of the Porcine IL-6 DuoSet ELISA Kit (DY686, R&D Systems, Minneapolis, MN) as described by Duarte et al. (2021). The concentration of IL-6 was described as ng/mg of protein. The concentration of IL-8 in jejunal mucosa was measured by following instructions of the Porcine IL-8/CXCL8 DuoSet ELISA Kit (DY535, R&D Systems) as described by Moita et al. (2021). The concentration of IL-8 was described as ng/mg of protein. The concentrations of TNF-α in mucosa were measured using the Porcine Immunoassay ELISA Kit (PTA00; R&D System Inc. Minneapolis, MN, USA), as described by Chaytor et al. (2011). The standard was used in a working range of 0 to 1,500 pg/mL. Sample, standard, and control (50 μL each) were added to microplate wells coated with capture antibody in conjunction with biotinylated antibody reagent. Detection occurred using HRP, TMB (3,3ʹ, 5,5″-tetramethylbenzidine) substrate, and a stop solution of 2 mol/L H_2_SO_4_. Absorbance was read at 450 nm and 550 nm and the TNF-α concentration in mucosa was expressed as pg/mL and pg/mg protein, respectively. Malondialdehyde concentrations in the mucosa were measured using the Thiobarbituric Acid Reactive Substance Assay Kit (STA-330, Cell Biolabs, San Diego, CA, USA). The MDA standard was used in a working range of 0 to 125 μmol/L. Aliquots of 100 μL of each standard and sample were pipetted into microcentrifuge tubes, followed by the addition of 100 μL of SDS lysis and incubation for 5 min at room temperature. Then, 250 μL of thiobarbituric acid reagent was added into each sample and standard, and incubated at 95 °C for 60 min in a water bath. All tubes were cooled to room temperature in an ice bath for 5 min before being centrifuged (10,000 × *g* for 15 min). Aliquots of 300 μL of supernatant were transferred to another tube with 300 μL of butanol and vortex for 1 min and centrifuge for 5 min at 10,000 × *g*. Aliquots of 200 μL of samples and standards were transferred to a 96-well microplate. The absorbance was measured at 532 nm, and the MDA concentration in the mucosa was expressed as μmol/mL and μmol/mg protein, respectively. The concentration of protein carbonyl was measured using the ELISA kit (STA-310, Cell Biolabs, San Diego, CA, USA) as described by Moita et al. (2021). Before measurement, mucosa samples were diluted in PBS to reach the protein concentration at 10 μg/mL. The standard was prepared by mixing the oxidized BSA and reduced BSA to reach the working range of 0.375 to 7.5 nmol/mg protein. Aliquots of 100 μL of standard or samples were added into the 96-well protein binding plate. The content of protein carbonyl in the samples and standard were derivated to dinitrophenyl (DNP) hydrazine and probed with an anti-DNP antibody, followed by incubation with a horseradish peroxidase (HRP) conjugated with the secondary antibody. The absorbance was measured at 450 nm, and the concentration of protein carbonyl was expressed as nmol/mg protein.

### Intestinal morphology and crypt cell proliferation

Two sections of jejunum per pig fixed in 10% buffered formalin were transferred to a 70% ethanol solution for 2 d and sent to North Carolina State University Histology Laboratory (College of Veterinary Medicine, Raleigh, NC, USA) for dehydration, embedment, staining and Ki-67 assay according to their internal standard protocol, for morphological evaluation and Ki-67 immunohistochemistry staining. The VH and CD were measured using a camera Infinity 2-2 digital CCD attached to a microscope Olympus CX31 (Lumenera Corporation, Ottawa, Canada). Lengths of 15 well-oriented intact villi and their associated crypts were measured in each slide. The villi length was measured from the top of the villi to the villi-crypt junction and the crypt depth was measured from the villi-crypt junction to the bottom of the crypt. Then, the VH:CD was calculated. Images of 15 intact crypts from each slide were cropped and the ImageJS software was used for calculating the ratio of Ki-67 positive cells to total cells in the crypt (%) as described by Baker et al. (2024). All analyses of the intestinal morphology were executed by the same person.

### Gene expression of tight junction proteins and MUC-1 and MUC-2

The RNA was extracted from mid-jejunal tissue following Jang et al. (2023a). Frozen mid-jejunal tissue (50 to 100 mg) was mixed in 1 mL tube with precooling Trizol reagent (#15-596-026, Invitrogen, Waltham, MA, USA) then the samples were processed at 4.5 m/s for 30 s two times using a Bead Mill 24 homogenizer (#15-340-163, Thermo Fisher Scientific Inc.). Homogenized samples were centrifuged at 12,000 × g for 10 min at 4 °C to get supernatants. 200 μL of chloroform (#146543, Thermo Fisher Scientific Inc.) was mixed with the supernatant in a new tube and incubated at room temperature for 10 min. After incubation, the mixed samples were centrifuged to get the aqueous phase and mixed with 200 μL of isopropanol (#B0518327, Acros Organics, Geel, NJ, USA). After 10 min resting, the mixed samples were centrifuged to get the sediment and then mixed with 75% ethanol. The mixed samples were centrifuged to remove the supernatants and then mixed with 40 μL DEPC water. The RevertAid First Strand cDNA Synthesis kit (#01299151, Thermo Fisher Scientific Inc.) was used to revert the extracted RNA into cDNA. All the procedures followed the manufacturer’s instructions. The CFX Connect Real-Time PCR Detection System (BioRad, Hercules, CA, USA) and Maxima SYBR Green/ROX qPCR Master Mix (#01292815, Thermo Fisher Scientific Inc.) were used for quantitative RT-PCR (qRT-PCR). The primers used for the tight junction proteins and mucin are listed in Table 2 and were synthesized by a commercial company (Millipore Sigma, Burlington, MA, USA). Delta–delta Ct values were calculated to get a relative expression of each target gene.

**Table 2.** Sequence of primers for tight junction proteins in jejunum of nursery pigs

Gene^1^	Primer sequences (5ʹ to 3ʹ)^2^	Accession number
ZO-1	F: CAGAGACCAAGAGCCGTCC	XM_003480423.4
	R: TGCTTCAAGACATGGTTGGC	
OC	F: TCAGGTGCACCCTCCAGATT	NM_001163647.2
	R: AGGAGGTGGACTTTCAAGAGG	
CL-1	F: ATTTCAGGTCTGGCTATCTTAGTTGC	NM_001244539.1
	R: AGGGCCTTGGTGTTGGGTAA	
GAPDH	F: TCGGAGTGAACGGATTTGGC	NM_001206359.1
	R: TGCCGTGGGTGGAATCATAC	
MUC-1	F: GGGGGACCGGTATAAAGCAG	XM_020945387.1
	R: CCCTTGTCATGATGGCGGTA	
MUC-2	F: CAACGGCCTCTCCTTCTCTGT	XM_021082584.1
	R: GCCACACTGGCCCTTTGT	

^1^ZO-1, zonula occludens; OC, occludin; CL-1, claudin-1; GAPDH, glyceraldehyde-3-phosphate dehydrogenase; MUC-1, mucin-1; MUC-2, mucin-2.

^2^F, forward; R, reverse.

### Statistical analysis

Data were analyzed based on a randomized complete block design using the MIXED procedure on SAS 9.4 (SAS Inc., Cary, NC, USA). Experimental unit was the pen. Factors (feed type and β-mannanase supplementation) and their interactions were evaluated as fixed effects and BW and sex blocks served as random effects. Statistical differences were considered significant with *P *< 0.05 and tendency with 0.05 ≤ *P* < 0.10. The microbiome data were tested for normal distribution with the UNIVARIATE (Shapiro–Wilk test), and the nonnormally distributed data were analyzed using the GLIMMIX procedure through Poisson distributions according to McMurdie and Holmes (2014).

## Results

### Digesta viscosity

Feeding LCF to nursery pigs did not affect the viscosity of jejunum compared to control. However, pigs fed LCF + tended to decrease (*P* = 0.088) digesta viscosity from the jejunum compared to LCF (Table 3).

**Table 3.** Digesta viscosity in the jejunum of nursery pigs fed control or LCF diets^1^ with or without β-mannanase

Diet^1^	Control	LCF			*P*-value	
M^2^	-	-	+	SEM	Diet	M
Viscosity, cP	2.45^ab^	2.95^a^	2.27^b^	0.26	0.184	0.088

^1^Control and low-cost formulated feed (LCF).

^2^Mannanase supplemented at 100 g/t, supplying 800 TMU/kg.

^ab^Within a row, values with different superscripts are significantly different at *P* < 0.05.

### Diversity and relative abundance of jejunal mucosa-associated microbiota

Feed type had no effect on α-diversity including Chao-1, Shannon, and Simpson indices of jejunal mucosa-associated microbiota at the species (Table 4). Pigs fed LCF + decreased (*P* < 0.05) Chao1 α-diversity but had no effect on Shannon or Simpson indices at the species level.

**Table 4.** Alpha-diversity of mucosa-associated microbiota at the species level in the jejunum of nursery pigs fed control or LCF diets^1^ with or without β-mannanase

Diet^1^	Control	LCF			*P*-value	
M^2^	−	−	+	SEM	Diet	M
Species						
Chao1	195.2	190.8	159.9	44.0	0.551	<0.001
Shannon	4.8	4.6	4.2	0.8	0.899	0.709
Simpson	0.9	0.9	0.8	0.4	0.964	0.946

^1^Control and low-cost formulated feed (LCF).

^2^Mannanase supplemented at 100 g/t, supplying 800 TMU/kg.

At the phylum level, LCF decreased (*P* < 0.05) the relative abundance of Firmicutes and increased (*P* < 0.05) Proteobacteria in the jejunal mucosa of nursery pigs (Table 5). Pigs fed LCF + increased (*P* < 0.05) the relative abundance of Firmicutes and decreased (*P* < 0.05) the relative abundance of Actinobacteria and Proteobacteria.

**Table 5.** Relative abundance of mucosa-associated microbiota at the phylum level in the jejunum of nursery pigs fed Control or LCF diets^1^ with or without β-mannanase

Diet^1^	Control	LCF			*P*-value	
M^2^	−	−	+	SEM	Diet	M
Firmicutes	52.1	41.0	59.7	7.6	0.008	0.002
Actinobacteria	25.2	22.9	17.6	5.9	0.373	0.038
Proteobacteria	14.3	16.3	11.3	11.2	0.003	<0.001
Bacteroidetes	0.9	1.9	2.7	0.6	0.134	0.301
Spirochaetae	0.0	0.1	0.4	0.2	0.587	0.272
Other	2.2	2.9	3.6	1.1	0.398	0.431

^1^Control and low-cost formulated feed (LCF).

^2^Mannanase supplemented at 100 g/t, supplying 800 TMU/kg.

At the family level, LCF decreased (*P* < 0.05) the relative abundance of Lactobacillaceae and increased (*P* < 0.05) the relative abundance of Helicobacteraceae (Table 6). Pigs fed LCF + decreased (*P* < 0.05) the relative abundance of Bifidobacteriaceae and Helicobacteraceae, increased (*P* < 0.05) the relative abundance of Streptococcaceae and Ruminococcaceae, and tended to increase (*P* = 0.061) Lachnospiraceae.

**Table 6.** Relative abundance of mucosa-associated microbiota at the family level in the jejunum of nursery pigs fed control or LCF diets^1^ with or without β-mannanase

Diet^1^	Control	LCF			*P*-value	
M^2^	–	−	+	SEM	Diet	M
Lactobacillaceae	39.1	29.1	27.3	5.7	0.006	0.536
Bifidobacteriaceae	23.4	21.1	16.3	5.6	0.364	0.047
Helicobacteraceae	12.3	18.7	8.6	10.8	0.003	<0.001
Streptococcaceae	3.3	2.7	17.8	3.2	0.473	<0.001
Lachnospiraceae	2.0	2.3	4.2	0.8	0.762	0.061
Veillonellaceae	1.8	1.8	2.4	0.5	1.000	0.476
Erysipelotrichaceae	2.6	1.9	2.2	0.6	0.360	0.712
Prevotellaceae	0.7	1.3	2.3	0.6	0.255	0.192
Staphylococcaceae	0.8	0.3	0.9	0.3	0.253	0.187
Ruminococcaceae	0.8	0.8	2.3	0.5	0.953	0.041
Coriobacteriaceae	1.2	1.0	0.7	0.4	0.725	0.612
Others	8.5	6.2	6.9	2.3	0.108	0.602

^1^Control and low-cost formulated feed (LCF).

^2^Mannanase supplemented at 100 g/t, supplying 800 TMU/kg.

At the genus level, LCF decreased (*P* < 0.05) the relative abundance of *Streptococcus, Staphylococcus,* and *Sharpea,* and increased (*P* < 0.05) *Helicobacter* (Table 7). Pigs fed LCF + increased (*P* < 0.05) the relative abundance of Lactobacillus and Streptococcus and decreased (*P* < 0.05) the relative abundance of *Bifidobacterium* and *Helicobacter.*

**Table 7.** Relative abundance of mucosa-associated microbiota at the genus level in the jejunum of nursery pigs fed control or LCF diets^1^ with or without β-mannanase

Diet^1^	Control	LCF			*P*-value	
M^2^	−	−	+	SEM	Diet	M
*Lactobacillus*	35.8	31.6	41.0	3.8	0.192	0.010
*Bifidobacterium*	22.7	23.8	17.9	3.1	0.681	0.027
*Helicobacter*	5.2	13.5	6.1	3.8	<0.001	0.001
*Streptococcus*	7.4	3.5	13.3	4.6	0.004	<0.001
*Staphylococcus*	2.7	0.1	0.8	0.4	0.012	0.122
*Sharpea*	2.9	1.1	1.4	0.9	0.040	0.655
*Syntrophococcus*	1.3	0.7	1.5	0.5	0.316	0.198
*Megasphaera*	0.8	1.0	1.2	0.4	0.767	0.711
*Olsenella*	0.8	0.5	1.3	0.3	0.544	0.169
*Mitsuokella*	0.6	0.4	0.9	0.3	0.542	0.251
*Corynebacterium*	0.4	0.1	0.5	0.2	0.257	0.203
Others	14.8	17.5	13.9	1.8	0.225	0.110

^1^Control and low-cost formulated feed (LCF).

^2^Mannanase supplemented at 100 g/t, supplying 800 TMU/kg.

At the species level, LCF increased (*P* < 0.05) *Helicobacter rappini, Bifidobacterium thermacidophilum-thermophilum, H. ganmani,* and *Lactobacillus johnsonii*, and decreased (*P* < 0.05) the relative abundance of *B boum, Sharpea azabuensis,* and *Staphylococcus saprophyticus-xylosus* (Table 8). Pigs fed LCF + increased (*P* < 0.05) the relative abundance of *B boum,* and tended to increase (*P* = 0.070) the relative abundance of *L delbrueckii-sp29223.* Additionally, LCF + decreased (*P* < 0.05) the relative abundance of *B thermacidophilum-thermophilum* and *H ganmani.*

**Table 8.** Relative abundance of mucosa-associated microbiota at the species level in the jejunum of nursery pigs fed control or LCF diets^1^ with or without β-mannanase

Diet^1^	Control	LCF			*P*-value	
M^2^	−	−	+	SEM	Diet	M
*Lactobacillus delbrueckii*	16.9	12.5	19.6	4.9	0.532	0.322
*Helicobacter rappini*	3.9	6.9	4.8	3.2	0.026	0.117
*Bifidobacterium boum*	14.2	5.9	10.3	1.9	0.001	0.011
*Bifidobacterium thermacidophilum-thermophilum*	8.7	18.1	7.8	1.6	0.001	<0.001
*Lactobacillus sp 29233*	4.7	5.9	6.6	1.3	0.364	0.604
*Helicobacter ganmani*	0.4	3.4	0.3	1.3	0.001	0.001
*Lactobacillus delbrueckii-sp29223*	3.7	2.5	4.4	1.3	0.217	0.070
*Lactobacillus* sp.	1.6	3.0	2.7	0.8	0.128	0.740
*Lactobacillus mucosae*	2.1	3.6	2.5	0.9	0.106	0.218
*Helicobacter equorum*	0.7	0.6	0.7	0.4	0.946	0.811
*Sharpea azabuensis*	2.9	1.1	1.4	0.9	0.040	0.655
*Syntrophococcus sp33249*	1.2	0.7	1.4	0.5	0.325	0.195
*Lactobacillus ruminis*	1.4	1.0	0.9	0.4	0.492	0.839
*Streptococcus* sp.	1.5	1.2	0.9	0.4	0.725	0.601
*Lactobacillus johnsonii*	0.5	2.1	1.5	0.7	0.020	0.408
*Megasphaera sp36946*	0.8	1.0	1.2	0.4	0.756	0.729
*Staphylococcus saprophyticus-xylosus*	1.6	0.1	0.3	0.3	0.040	0.396
*Staphylococcus saprophyticus*	0.8	0.0	0.2	0.2	0.397	0.557
Others	26.4	25.5	26.2	6.8	0.748	0.816

^1^Control and low-cost formulated feed (LCF).

^2^Mannanase supplemented at 100 g/t, supplying 800 TMU/kg.

### Intestinal oxidative stress and inflammatory status, morphology and crypt cell proliferation, and gene expression of tight-junction proteins and MUC-1 and -2

The concentration of pro-inflammatory cytokines (IL-6, IL-8, and TNF-α) and oxidative damage product, protein carbonyl, were not affected by diet type or β-mannanase supplementation, however, LCF tended to decrease (*P* = 0.097) MDA concentrations in the jejunum (Table 9).

**Table 9.** Immune parameters, oxidative stress, morphology, crypt cell proliferation, and expression of tight junction protein genes in the jejunum of nursery pigs fed control or LCF diets^1^ with or without β-mannanase

Diet^1^	Control	LCF			*P*-value	
M^2^	−	−	+	SEM	Diet	M
Inflammation and oxidative stress^3^						
IL-6, pg/mg	10.46	12.66	10.58	1.98	0.239	0.324
IL-8, ng/mg	0.45	0.43	0.45	0.02	0.504	0.502
TNF-α, pg/mg	2.02	2.20	2.19	0.32	0.543	0.915
MDA, nmol/mg	0.30^a^	0.20^b^	0.27^ab^	0.04	0.097	0.232
PC, nmol/mg	1.18	1.02	1.07	0.14	0.380	0.766
Morphology and proliferation^3^						
VH, µm	457.3	447.4	474.1	26.4	0.794	0.486
CD, µm	199.1	210.2	213.9	11.2	0.497	0.818
VH:CD	2.30	2.13	2.22	0.11	0.134	0.379
Ki-67^+^, %	18.50	21.63	20.82	2.02	0.220	0.742
Genes^4^						
ZO-1	1.09^a^	0.76^b^	0.84^ab^	0.13	0.098	0.721
OC	0.91	0.75	0.91	0.09	0.249	0.223
CL-1	1.45	0.98	1.33	0.65	0.309	0.236
MUC-1	1.03	1.08	1.14	0.27	0.921	0.873
MUC-2	1.06	1.41	1.29	0.20	0.233	0.692

^1^Control and low-cost formulated feed (LCF).

^2^Mannanase supplemented at 100 g/t, supplying 800 TMU/kg.

^3^Ki-67^+^, ratio of Ki-67-positive cells to total cells in the crypt.

^4^ZO-1, zona occludens; OC, occuldin; CL-1, claudin-1; MUC-1, mucin-1; MUC-2, mucin-2.

^ab^Within a row, values with different superscripts are significantly different at *P* < 0.05.

Diet type and β-mannanase supplementation had no effect on VH, CD, VH:CD, or Ki-67^+^ cells in the crypt.

Pigs fed LCF tended to decrease (*P* = 0.098) the gene expression of ZO-1 in the jejunum, however, had no effect on the expression of OC, CL-1, MUC-1, or MUC-2. Pigs fed LCF + had no effect on the gene expression of OC, CL-1, MUC-1, or MUC-2 however, increased gene expression of ZO-1 to a level that was no longer statistically different from the control.

### Growth performance and fecal score

No effects were observed for diet type or β-mannanase supplementation on BW on days 0, 12, and 21 however, LCF tended to decrease (*P* = 0.090) BW at day 37 whereas LCF + tended to increase (*P* = 0.059) BW at day 37 (Table 10). Additionally, LCF decreased ADFI (*P* < 0.05) during phases 1, 3, and for the overall period and tended to decrease (*P* = 0.084) ADG for the overall period. Pigs fed LCF + tended to increase (*P* = 0.056) ADFI during phase 1, increased (*P* < 0.05) ADG during phase 3, and tended to increase (*P* = 0.054) ADG for the overall period. No effects were observed on G:F for LCF during any of the phases, however, LCF + increased (*P* < 0.05) G:F during phase 3 and for the overall period. Moreover, pigs fed LCF + decreased (*P* < 0.05) feed cost per kg body weight gain during phase 3, whereas diet type had no effect during any phase of the study. No effects of diet were observed on fecal score during phase 1 or 2 however, LCF increased (*P* < 0.05) fecal score during phase 3. Pigs fed LCF + tended to decrease (*P* = 0.080; *P* = 0.067) fecal scores during phases 1, and 2, and decreased (*P* < 0.05) fecal scores during phase 3.

**Table 10.** Growth performance and fecal score of nursery pigs fed control or LCF diets^1^ with or without β-mannanase

Diet^1^	Control	LCF			*P*-value	
M^2^	–	−	+	SEM	Diet	M
BW, kg						
Day 0	6.2	6.2	6.1	0.4	0.800	0.800
Day 12	8.3	7.6	8.0	0.6	0.110	0.279
Day 21	12.2	11.4	12.1	0.9	0.327	0.398
Day 37	24.8	22.7	25.1	1.2	0.090	0.059
ADG, g/d						
Phase 1^3^	178	118	158	24	0.106	0.270
Phase 2	437	422	450	41	0.825	0.660
Phase 3	788^ab^	707^b^	812^a^	34	0.279	0.025
Overall	505^a^	446^b^	512^a^	24	0.084	0.054
ADFI, g/d						
Phase 1	250^a^	164^b^	224^a^	22	0.009	0.056
Phase 2	635	569	612	49	0.308	0.507
Phase 3	1,340^a^	1,180^b^	1,196^b^	60	0.026	0.831
Overall	815^a^	701^b^	739^ab^	40	0.027	0.455
G:F						
Phase 1	0.69	0.67	0.73	0.09	0.921	0.687
Phase 2	0.69	0.74	0.74	0.04	0.431	0.937
Phase 3	0.59^a^	0.60^a^	0.69^b^	0.03	0.548	0.048
Overall	0.62^a^	0.64^a^	0.70^b^	0.02	0.717	0.021
Feed cost/kg BW gain, $						
Phase 1	1.41	1.13	1.27	0.14	0.191	0.500
Phase 2	0.98	0.83	0.87	0.06	0.118	0.639
Phase 3	0.81	0.75	0.66	0.03	0.167	0.022
Fecal score						
Phase 1	3.9^ab^	4.0^a^	3.7^b^	0.1	0.708	0.080
Phase 2	3.8^a^	3.8^a^	3.5^b^	0.1	0.889	0.067
Phase 3	3.3^a^	3.4^b^	3.1^c^	0.1	0.002	<0.001

^1^Control and low-cost formulated feed (LCF).

^2^Mannanase supplemented at 100 g/t, supplying 800 TMU/kg.

^3^Phase 1, days 0 to 12; phase 2, days 12 to 21; phase 3, days 21 to 37.

^abc^Within a row, values with different superscripts are significantly different at *P* < 0.05.

## Discussion

Various factors, such as housing patterns, feedstuffs, and ages could influence the efficacy of β-mannanase on immune response, oxidative stress, and morphology in the jejunum of nursery pigs. Previous studies showed that group housing could affect the physiological response, behaviors, and intestinal immune response, and intestinal microbiota of pigs (Bruininx et al., 2002; Wen et al., 2021). In the current study, pigs were housed individually to obtain the β-mannanase intake in individual pigs in order to relate its effects on mucosa-associated microbiota, immune response, oxidative stress, and morphology in the jejunum collectively influencing growth performance of nursery pigs as suggested by previous studies (Deng et al., 2022; Choi et al., 2024; Jang et al., 2024). Increased digesta viscosity in nursery pigs has been associated with decreases in nutrient digestibility (Moita et al., 2022; Baker et al., 2024) and exacerbation of postweaning diarrhea (Hopwood et al., 2004), and may increase the rate of villus atrophy (Hedemen et al., 2006). In the present study, β-mannanase supplementation tended to decrease the digesta viscosity in the jejunum of nursery pigs which may have enhanced the absorption of nutrients to be used for growth, whereas limiting the amount of unabsorbed nutrients for potentially pathogenic microorganism proliferation. The results in this study are in agreement with Jang et al. (2024), as digesta viscosity in the jejunum of nursery pigs was decreased with β-mannanase supplementation.

Furthermore, LCF + decreased the Chao1 α-diversity of jejunal mucosa-associated microbiota at the species level. Lower diversity is typically perceived as negative as some researchers point to the association between reduced microbial diversity and disease, highlighting that a species-rich ecosystem may be more robust against environmental stressors (Valdes et al., 2018). Recent studies have shown higher levels of dietary fiber can result in decreased α-diversity in healthy populations (Clarke et al., 2017; An et al., 2019; Reider et al., 2020). Furthermore, Fu et al. (2022) hypothesized this effect may be due to diets higher in fiber leading to the enrichment of specific fiber-digesting strains of bacteria, most of which are beneficial short-chain fatty acid producers, inhibiting the residence or growth of other species, thereby eliciting a temporary loss of alpha diversity (Valdes et al., 2018). Indeed, the LCF feed had increased levels of SBM and thus increased NSP compared to the control, which contained hydrolyzed soybean meal and increased levels of fish meal in place of SBM. Additionally, on a basic level, the LCF feed simply had a lower number of feedstuffs included in formulation thus providing a relatively lower number of different substrates available for highly specialized species of microbiota in the intestinal tract. In agreement with Adhikari et al. (2019), Firmicutes were the predominant phylum observed in the jejunal mucosa of nursery pigs among all treatments in the current study. The LCF treatment, however, decreased the relative abundance of Firmicutes and increased Proteobacteria. The phylum Proteobacteria contains many gram-negative bacteria including *Escherichia*, *Campylobacter*, *Salmonella*, *Vibrio*, and *Helicobacter*, and overgrowth of these microbes can disturb the intestinal barrier function and consequently result in enteric diseases (Zhang et al., 2018). At the genus level, LCF+ increased the relative abundance of *Lactobacillus* and *Streptococcus* and decreased the relative abundance of *Helicobacter.* The most commonly used species of probiotics are strains of lactic acid bacteria in genera such as *Lactobacillus, Bifidobacterium,* and *Streptococcus* (Patil et al., 2015), as these species resist bile acid and possess the ability to colonize the intestine and competitively exclude potentially pathogenic bacteria (Verdenelli et al., 2009). These probiotics are commonly distinguished as conferring the growth of a healthy microbiome (Walter et al., 2008), reducing intestinal colonization of enteric pathogens (Huang et al., 2004; Lee et al., 2012), and improving digestive ability and antibody-mediated immune responses (Wang et al., 2012; Hou et al., 2015). *Helicobacter* spp. such as *H. rappini* have been shown to translocate in enterocytes and cause bacteremia (Chalifoux et al., 1985; Fox, 2002) which may stimulate the systemic immune response, further restricting nutrient utilization for growth. Other *Helicobacter* spp. such as *H. ganmani* have been associated with an increase in pro-inflammatory cytokine IL-12, and until further characterization, is regarded as a potential pathogen (Alvarado et al., 2015). Pigs fed LCF+ reduced the relative abundance of *H. rappini*, *H. ganmani*, and their associated genus (*Helicobacter*) and family (Helicobacteraceae).

The intestinal tract is in constant contact with foreign substances and selectively absorbs nutrients whilst neutralizing or eliminating toxins and enteric pathogens (Tang et al., 2016; Duarte et al., 2023). Several defense mechanisms are provided by the epithelium of the intestinal tract, one of which is the establishment of a permeability barrier, regulated by tight junction proteins (Turner, 2009). Tight junction proteins consist of numerous intracellular and apical intercellular membrane proteins that regulate the ion selectivity and pore size of the epithelium such as zonula occludens, occludin, and claudins (Edelblum and Turner, 2009; Groschwitz and Hogan, 2009; Marchiando et al., 2010). In the present study, LCF tended to decrease the expression of ZO-1 gene in the jejunum of nursery pigs, whereas there was no effect of β-mannanase on the expression of ZO-1 genes comparing between LCF+ and LCF. However, it is interesting to note the expression of ZO-1 gene in LCF+ was not different than Control. In addition, oxidative stress products and inflammatory cytokines in the jejunal mucosa showed no difference between LCF + and Control. Intestinal morphology is also highly related to barrier functions (Wijtten et al., 2011). In this study, no statistical difference was observed in villus height between LCF+ and Control. These results partly indicate slight improvements in barrier function with β-mannanase supplementation to LCF feeds.

In the present study, LCF reduced ADFI during phases 1, 3, decreased ADFI, ADG, and BW for the overall period, and increased fecal score during phase 3. This result could be explained by the high level of SBM included in these diets relative to the control. Reductions in ADFI, ADG, and BW have been observed with high inclusion of soy products including raw soybeans (Yen et al., 1977), heated SBM (Zarkadas and Wiseman, 2005), and low trypsin inhibitor SBM (Cook et al., 1988). Observed improvements in the current study on growth responses with β-mannanase supplementation are in agreement with Yoon et al. (2010) and Jang et al. (2024), as LCF+ increased ADFI during phase 1, and increased ADG, BW, and G:F for the overall period. Interestingly, in a recent meta-analysis on β-mannanase supplementation in pigs by Kipper et al. (2020), the author reported improvements in BW gain and feed efficiency were observed in 81 and 83% of studies with β-mannanase supplementation compared to the controls. This effect could be due to the hydrolysis of β-mannan by β-mannanase that could otherwise cross the intestinal mucosa and trigger an innate immune-system response (Jackson et al., 2003). This feed-induced immune response consumes energy which could otherwise be used for animal growth during a normal metabolic state (Ferreira et al., 2016). Additionally, β-mannan has been associated with reduced nutrient absorption, increased digesta viscosity and hormonal changes in previous publications (Shastak et al., 2015). Reductions in nutrient absorption may be due to the interaction between β-mannan and glycocalyx, which leads to mucus layer thickening and physical prevention of the absorption of nutrients (Montanhini Neto et al., 2013). Increased amounts of unabsorbed nutrients in the intestinal lumen creates a favorable environment for microorganism proliferation, some of which may be pathogenic and suppress performance by reducing health status (Teirlynck et al., 2009). Previous studies indicate that β-mannanase supplementation can also stimulate the activity of other digestive enzymes such as amylases and trypsin, further improving nutrient digestion and absorption in monogastric species (Li et al., 2010; Jang et al., 2023b). Additionally, increased amounts of SBM in feeds have been shown to favor the occurrence of diarrhea in nursery pigs (Dréau et al., 1994) due to the presence of anti-nutritional compounds that stimulate a local intestinal immune response (Fairbrother et al., 2005). Indeed, in the present study LCF+ decreased digesta viscosity, had favorable effects on the mucosa-associated microbiota, improved growth performance, and decreased fecal score during phase 3. The improvements observed in the present study with β-mannanase supplementation are also reflected in the cost per kg of BW gain as LCF + decreased cost per kg of BW gain during phase 3 by 12% ($0.75/kg BW gain to $0.66/kg BW gain) compared to LCF, and 18.5% ($0.81/kg BW gain to $0.66/kg BW gain) compared to Control.

The animal protein supplements are widely utilized in nursery pig feeds to ease the transition from sow’s milk to solid food, however, prices and availability for these feedstuffs have become extremely volatile in recent years which could increase the overall cost of pork production and place increased pressure on finding alternatives in the case of limited supply or availability. Due to the transitory hypersensitivity reaction to SBM mainly induced by allergenic proteins and indigestible NSP such as β-mannan, SBM is typically limited in the early postweaning period. In the future, SBM may become cheaper as government incentives for renewable diesel favor the increased crushing of soybeans for extraction of oil thus resulting in increased amounts of SBM available for procurement. Pig producers could take advantage of the decreased cost of SBM and increase the inclusion level in nursery pig diets if growth and health are not impacted.

In the present study, increased amounts of SBM in LCF increased the relative abundance of potentially pathogenic microbiota, reduced the expression of ZO-1 genes, and decreased growth performance. Pigs fed LCF + showed decreased jejunal digesta viscosity, increased relative abundance of *Lactobacillus*, decreased *Helicobacter*, returned tight junction gene expression of ZO-1 to levels comparable to the Control, enhanced growth performance, decreased feed cost per kg of BW gain, and decreased fecal score of nursery pigs, indicating the efficacy of β-mannanase in feeds utilizing high amounts of SBM.

## Abbreviations

ADFI: average daily feed intake
ADG: average daily gain
BW: body weight
CD: crypt depth
cDNA: complementary DNA
CL-1: claudin-1
cP: centipoise
DNA: deoxyribonucleic acid
DNP: dinitrophenyl
FTU: phytase unit
GAPDH: glyceraldehyde-3-phosphate dehydrogenase
G:F: gain to feed ratio
HRP: horse radish peroxidase
IL-8: interleukin-8
IL-6: interleukin-6
LCF: Low-cost formulation
MAMPs: molecular associated microbial patterns
MDA: malondialdehyde
ME: metabolizable energy
MUC-1: mucin-1
MUC-2: mucin-2
NSP: nonstarch polysaccharide
OC: occuldin
OTU: operational taxonomic units
PBS: phosphate-buffered saline
PC: protein carbonyl
PRRs: pathogen recognition receptors
RNA: ribonucleic acid
RT-qPCR: Real-time quantitative real-time polymerase chain reaction
SBM: soybean meal
SID: standardized ileal digestible
TMU: thermostable mannanase unit
TNF-α: tumor necrosis factor alpha
VH: villus height
ZO-1: zona occluden 1
